# Beyond the Screen: Medical Education in the Era of Artificial Intelligence

**DOI:** 10.1007/s40670-026-02888-0

**Published:** 2026-09-28

**Authors:** Andrea Dean, Geeta Singhal

**Affiliations:** Department of Pediatrics, Pediatric Hospital Medicine, Baylor College of Medicine, Texas Children’s Hospital, 1102 Bates Street, Suite FC 1860, Houston, TX 77030 United States

**Keywords:** Electronic medical record (EMR), Bedside teaching, Cognitive load, Artificial intelligence, Physician–patient relationship

## Abstract

“Screen retreat,” where trainees instinctively turn to the electronic medical record (EMR) rather than the bedside when questions arise, has overtaken medical education. While often justified as efficient, this reliance displaces patient interaction, increases cognitive burden, and offers a psychological refuge from the uncertainty of direct care, where challenge leads to learning. As a result, trainees miss out on developing skills in communication, empathy, and clinical judgment. In the era of artificial intelligence, this shift risks further diminishing the need for human physicians. Medical education must urgently be recentered on bedside engagement and the intentional cultivation of uniquely human capacities.

As we left the patient’s room, I asked the resident, “Remind me, is this child on an asthma controller?” “Oh!” he replied enthusiastically. “I’ll find out.” But instead of turning around to clarify with the family, he hurried to the computer down the hall and fumbled through the electronic medical record (EMR). 

The scene is familiar in modern medical training. Trainees instinctively retreat to screens when new information emerges, care plans change, or teaching moments arise. Reliance on digital tools is often justified as modern and efficient, but in reality, it is often slower and less reliable. More importantly, because trainees have finite time in the clinical environment, accumulating digital demands and screen retreat come at the expense of patient interaction and bedside learning. 

As such, screen retreat threatens the heart of medical education. When trainees turn first to the EMR rather than to patients, families, and interprofessional colleagues, they lose opportunities for experiential learning [1], situated learning [2], and professional identity formation [3] at the bedside. These are not abstract educational ideals; these foundational theories of learning affirm that medical education depends on the daily, often silent, processes of authentic clinical participation and development within communities of practice. Through these processes, trainees not only acquire knowledge, but internalize the values, behaviors, and identity of the profession that will define clinical care for the next generation of physicians. 

This erosion of bedside learning does not argue for the rejection of technological advances in medicine. Quite the opposite. Precisely because these advances are both inevitable and full of promise, medical education must be deliberate about protecting the analog foundations of doctoring and ensuring that digital tools support, rather than replace, bedside learning. As we embrace artificial intelligence (AI), we must make the human contribution to patient care obvious and undeniable by creating clinicians who are compassionate, expert communicators, and capable of complex judgment, skills that can only be developed through direct patient care.

## Charting without Connection

While evidence supports the increased burden of documentation [4], it likely accounts for only a small portion of current screen time in our trainees. Instead, the real problem of time spent is that where charting was once a valuable exercise in synthesizing data and expressing clinical reasoning, it is now mostly a legal and compliance-driven task with diminishing educational value, dominated by billing requirements, compliance demands, and duplication. As such, hours spent behind the screen for documentation away from patients are lost bedside opportunities with little learning benefit.

## Tasks without Patients

Another significant and insufficiently studied contributor to screen retreat is secure messaging and task overload. Trainees face a flood of EMR messages, texts, and pages from nurses, consultants, and other staff. Even a brief absence from the computer creates a backlog of tasks that must be addressed upon the trainee’s return. While resident-specific data are limited, studies of physicians indicate that higher messaging volume correlates with greater EMR time and more frequent attention-switching, a marker of cognitive burden [5]. Trainees are especially vulnerable to the cognitive overload that results from this phenomenon, because they do not yet have the experience or knowledge to effectively triage incoming tasks. They also may worry about being evaluated by supervisors or reported by staff if their response is delayed. This pressure to catch up may drive trainees to remain anchored to screens, leaving little time for spontaneous bedside interaction. Medical education must confront this burden directly. If left unchecked, digital task overload risks becoming the norm in the clinical learning environment, displacing the essential work of learning patient care through patient care.

## The Psychology of Retreat

A less visible but perhaps the most consequential driver of screen retreat is psychological. We propose that the EMR may function as a “security blanket” for trainees: a predictable, controlled environment that offers refuge from the uncertainty, visibility, and emotional demands of direct patient care. Direct patient interaction is high-stakes and unpredictable, requiring trainees to think and respond by integrating clinical knowledge, observation, and judgment in real time. In contrast, the EMR provides a familiar, self-paced environment that may offer emotional relief from the stress of bedside care. Additionally, compared with the open-ended nature of bedside engagement, screen-based work may feel more productive and controlled as trainees simultaneously check off administrative tasks and answer messages.

Research on family-centered rounds illustrates this tension. Residents find presenting in front of families awkward or intimidating, even when they recognize its benefit for patients [6]. Fear of being wrong or revealing knowledge gaps may contribute to a preference for table rounding, where errors feel less visible and the demands of the EMR can be addressed concurrently. While it is widely agreed that psychological safety is crucial in the clinical learning environment, medical educators also agree that scaffolded challenge and discomfort, or Bjork’s concept of desirable difficulties, build judgment, confidence, and professional growth [7]. As such, we must combat trainees’ screen retreat and instead actively encourage them to face the uncertainty that comes with direct patient interaction.

## The AI Imperative

For practicing physicians, clerical burden and the electronic environment have been associated with burnout and lower professional satisfaction, in part because they alter the physician-patient interaction and distract from the meaningful aspects of medical practice [8]. We argue that medical education faces a parallel risk: when screen-based work displaces direct patient interaction, trainees have fewer opportunities to practice the communication, empathy, and relational judgment that make doctoring distinctly human. Indeed, data show that graduates report feeling unprepared to handle core communication scenarios and perceive gaps in their training around psychosocial dynamics [9]. Additionally, some data suggest a decline in empathy as medical trainees advance in their education [10].

Meanwhile, the shifting landscape of clinical care puts physician jobs at risk. AI is increasingly capable of performing tasks that once defined medical expertise: gathering data, identifying patterns, and generating care plans. The EPOCH score, an emerging framework proposed to measure the likelihood a job can be automated, captures five human-intensive capabilities: Empathy, Presence, Opinion, Creativity, and Hope [11]. Higher EPOCH scores correspond to roles rich in human interaction, judgment, and adaptability, while lower scores correspond to tasks more easily automated by AI. How we educate physicians now will help define the future of the physician role. If training remains centered on screen-facing tasks, it risks training future physicians toward the aspects of the role most vulnerable to automation, rather than enriching the distinctly human capacities captured by the EPOCH framework.

Medical education must be re-centered on cultivating uniquely human skills. The EPOCH score can provide a useful framework for designing curricula and assessment methods that emphasize bedside intuition, empathy, patient teaching, and real-time decision-making. These skills cannot be learned behind a screen or while EMR tasks define clinical service. Medical education must be intentional, protecting time for direct patient interaction, mentoring residents through high-stakes encounters, and modeling human engagement as a core tenet of doctoring.

## Recommendations to Reclaim Bedside Learning and Foster Human-Centered Skills

- Rapidly adopt AI tools in the clinical setting both to free trainees from administrative tasks and to allow a refocus on educational activities. For example, AI can complete compliance and billing-driven documentation, while trainee documentation is a pedagogical and assessment tool.
- Study and monitor the impact of secure messaging and other administrative tasks on cognitive load, workflow, and wellbeing specifically in medical trainees, and quantify the impact of face-to-face patient interaction on educational outcomes.
- Limit unnecessary computer use during teaching rounds. The role of trainees on rounds is to present a synthesis of data and clinical reasoning, not to read the EMR. If “work rounds” are required during high-census times, prioritize time-sensitive orders and tasks, such as consults and imaging, rather than administrative work.
- Emphasize patient interaction and full team engagement at the bedside on rounds. Each patient is an opportunity for all trainees to learn with and from patients, families, and their peers.
- Encourage true family-centered rounds or other opportunities for trainees to lead high-stakes discussions with patients and families. Provide mentoring to guide trainees through these encounters, priming them to distinguish comfort from psychological safety.
- Reassign previously outsourced patient care interactions to trainees. Tasks such as suicide screening, smoking-cessation counseling, or bedside discharge education are essential for bedside learning. Protect resident time for these activities by outsourcing screen-facing administrative tasks.
- Model bedside reassessment and follow-up whenever new information arises. Verbalize directions to trainees to personally communicate changes or new orders to patients and families, normalizing direct patient engagement as a core component of clinical care and education.
- Teach intentional technology use as a clinical skill. Trainees need explicit coaching to triage digital tasks, use EMR and AI tools critically, and recognize when bedside or team-facing communication is the better source of information.

Technology will transform medicine. Rapid integration of AI will clarify the present and future landscape of clinical care to enable necessary educational reform. The future of physicians depends on our ability to preserve and cultivate the human skills that AI cannot replicate. Medical education must center on bedside engagement, relational judgment, and empathy, with the goal of ensuring that physicians remain indispensable.

## Data Availability

Not applicable. No datasets were generated or analyzed for this commentary.
